# Optimization of Physical and Nutritional Parameters for Hyaluronidase Production by *Streptococcus mitis*

**DOI:** 10.4103/0250-474X.45412

**Published:** 2008

**Authors:** S. Sahoo, P. K. Panda, S. R. Mishra, A. Nayak, S. K. Dash, P. Ellaiah

**Affiliations:** * University Department of Pharmaceutical Sciences, Utkal University, Bhubaneswar-751 004, India; 1 P.G. Department of Microbiology, Orissa University of Agriculture and Technology, Bhubaneswar-751 003, India; 2 Pharmaceutical Biotechnology Division, Department of Pharmaceutical Sciences, Andhra University, Visakhapatnam-530 003, India

**Keywords:** Hyaluronidase, nutritional parameters, submerged fermentation, *Streptococcus mitis* MTCC*2695

## Abstract

The effect of some physical and nutritional parameters were studied for the optimum production of extracellular enzyme hyaluronidase employing *Streptococcus mitis* MTCC*2695 by submerged fermentation. The effects of initial pH, incubation temperature and time, inoculum level and age of inoculum were studied. The maximum enzymatic activity was obtained with an initial pH 5.8, incubation temperature 37°, incubation time for 48 h and inoculum level 6% with inoculum age 24 h. The effect of different carbon and nitrogen sources and antibiotics were studied. The results indicated that sucrose and ammonium chloride showed the highest enzymatic activity among various carbon and nitrogen sources. Antibiotic clarithromycin showed strong inhibitory effect on hyaluronidase production.

The therapeutic benefit of hyaluronidases (hyase) is based on the cleavage of hyaluronan in tissues resulting in increased membrane permeability, a reduced viscosity and a facilitated diffusion of injected fluids (referred to as spreading effect of hyases). The recombinant enzyme acts as an adjuvant; accelerates and increases absorption and dispersion of injected drugs, e.g. antibiotics, promotes resorption of excess fluids and improves the effectiveness of local anaesthesia^1^, for hypodermoclysis and as an adjunct in subcutaneous urography for improving resorption of radiopaque agents^2^. Bacterial hyaluronate lyases are considered as virulence factors that facilitate the spreading of bacteria in host tissues by degradation of hyaluronan^3^. Hyase facilitates diffusion of antiviral drugs, dyes and toxins^4^. Hyases, especially bovine testicular hyaluronidase (BTH) preparations, are widely used in many fields like orthopaedics, surgery, dentistry^5^, ophthalmology^6^ (vitrectomy), internal medicine, oncology^7^, dermatology and gynecology^8^. Testicular hyases have significant homology with the protein pH-20 (64 kD) present on the posterior head and the acrosomal membrane of mammalian sperm that plays an essential role in fertilization^9^. Based upon the medical, physiological, biological and commercial importance of hyases the present work was undertaken to optimize enzyme production parameters including effect of pH^10^, temperature, incubation period, inoculum level and age of inoculum employing *Streptococcus mitis* MTCC*2695. The effect of carbon source, inorganic nitrogen source and antibiotics on enzyme production was also studied.

A microbial strain, *Streptococcus mitis* MTCC*2695 procured from IMTECH Culture Collection Centre and Gene Bank, Chandigarh was used in the present study. It was rejuvenated by subculturing onto trypticase soy agar (TSA) plates with 5% defibrinated sheep blood (Imgenex Co., Bhubaneswar). The medium contains (g/l) trypticase soy broth, 30; and Agar, 15. The plates were incubated at 37° for 48 h. Small quantities of this culture was transferred onto nutrient agar slants and incubated at 37° for 24 h. The growth content of each slant was suspended in 5 ml of sterile water and the optical density (OD) of the pooled suspension was measured at 675 nm resulting 0.580 OD (equivalent to 1.01×10^6^ cfu/ml) that constitutes the inoculum. Inoculum level (5%) was transferred into 250 ml Erlenmeyer flask containing 50 ml of modified nutrient broth with composition (g/l) peptic digest of animal tissue, 5; sodium chloride, 5; beef extract, 1.5; yeast extract, 1.5; casein enzyme hydrolysate type-1, 4; KH_2_PO_4_, 3; magnesium sulphate, 3; hyaluronic acid (HA), 0.001% with pH 5.8.

After inoculation, the flasks were incubated at 37° on a rotary shaker (Ilshin Lab Co., Korea, Model BBT-1) at 150 rpm for 48 h. During fermentation, the microbial growth and hyase production were monitored. The microbial growth was monitored by measuring OD at 675 nm with UV-Visible spectrophotometer (Systronics, Model-118). At the end of fermentation 5 ml broth was aseptically withdrawn and centrifuged at 8000×g for 30 min at 4°. The clear supernatant was subjected to enzyme assay.

Hyase activity was measured spectrophotometrically by turbidity reduction assay^11^ using HA sodium salt from *Streptococcus equi* (Sigma Aldrich, USA) as a substrate. The enzymatic assay is based on Dorfmans method^12^. The enzymatic reduction in turbidity was measured after addition of 1 ml of HA at 70 μg/ml into 1 ml of enzyme sample solution in the presence of 0.05 M sodium phosphate buffer with 0.05 M NaCl (pH 7.0) and the resulting mixture was incubated for 30 min. To the above incubated mixture, 2.5 ml of acidified protein solution (1% w/v) bovine serum albumin fraction-V (BSA) in 0.5 M sodium acetate buffer, (pH 3.1) was added and incubated at 37° for 10 min and reduction in turbidity was read by measuring the absorbance at 600 nm. One unit of enzyme activity was defined as the amount of enzyme that reduced the absorbance by 0.1 at 600 nm (A_600_) in 30 min at 37°, pH 7.0 under assay conditions similar to that caused by one unit of an international standard.

To investigate the influence of initial pH on enzyme production, the production medium was adjusted to various levels of pH (4.0-9.0). Fermentation was conducted and samples were assayed for enzymatic activity. To study the effect of initial temperature and incubation period on enzyme production and cell growth, the production medium was inoculated and incubated at various temperatures ranging from 20° to 55° for 96 h. The samples were withdrawn at regular interval of 12 h and assayed for biomass (mg/ml) and enzymatic activity. The optimal temperature and incubation period obtained at this level was used for further studies. The flasks with the basal production medium were inoculated with inoculum age of 24 h level at 0.1, 1, 2, 4, 6 and 10% level and incubated at 37° for 48 h and 5ml samples were withdrawn at 12 h intervals and examined for biomass (mg/ml) and enzyme activity. The optimal level of inoculum obtained was used in further experiments.

The effect of various carbohydrates such as glucose, lactose, sucrose, mannitol, dextrin, dextrose, starch, sodium CMC and sodium alginate were studied by adding at a concentration of 5 mg/ml to the basal production medium^13^. Various inorganic nitrogen sources ammonium acetate, ammonium bicarbonate, ammonium chloride, ammonium sulphate, sodium nitrite and sodium nitrate were added (5 mg/ml) to the basal production medium and their effects were evaluated.

Different antibiotics clarithromycin, azithromycin, penicillin, gentamicin, cefixime and cefuroxime were added (10 μg/ml) to the basal production medium and assayed for enzyme content after fermentation. The above antibiotics were also tested for their activity against *S. mitis* MTCC*2695 by disc diffusion method^14^ and the zone of inhibition were recorded.

The results on the effect of initial pH on enzyme activity indicated that the highest enzyme yield was observed at pH 5.8 (181 U/ml) while the lowest was recorded at pH 9.0 (16 U/ml). There was a gradual decrease in enzyme yield from pH range 5.8 to 7.2, above and below this range, activity decreased sharply. The result of incubation period on the fermentation cycle is given in fig. 1. The highest enzyme activity (185 U/ml) and cell mass (3.6 mg/ml) at 48 h while the pH changed from 5.8 to 6.1.

**Fig. 1:**
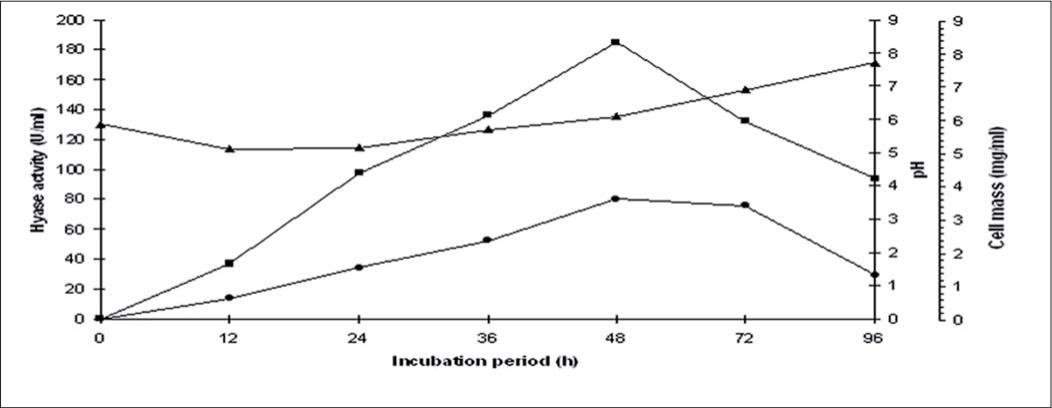
Time course profiles of hyase production and cell mass. Time course profiles of hyase production, cell mass (mg/ml) and pH by *S. mitis*. Enzyme activity (–■–), cell mass (–●–) and pH (–▲–).

The results also indicated that the optimum enzyme activity (179 U/ml) was obtained at 37° while a gradual decrease in enzyme activity was observed later. Further, the effect of inoculum size on hyase production is such that the highest enzyme activity (179 U/ml) and cell mass (3.3 mg/ml) was recorded with 6% inoculum where as the lowest yield (79 U/ml) and cell mass (0.83 mg/ml) was observed with 0.1% level. There was a gradual decrease in yield beyond 6% inoculum. This study also showed maximum enzyme production of 245, 210 and 189 U/ml with sucrose, dextrose and glucose, respectively. The other carbohydrates under test decreased the enzyme yield.

The effect of various inorganic nitrogen sources on enzyme production suggested that ammonium chloride gave the highest enzyme production (225 U/ml) followed by ammonium sulphate. The lowest yield was observed with sodium nitrate (103 U/ml). Among the different antibiotics clarithromycin exhibited the highest inhibitory activity (91%) followed by azithromycin where as the lowest inhibitory activity was recorded in penicillin (47%).
